# The impact of an exercise training intervention on cortisol levels and post-traumatic stress disorder in juveniles from an Ugandan refugee settlement: study protocol for a randomized control trial

**DOI:** 10.1186/s13063-018-2753-x

**Published:** 2018-07-09

**Authors:** Henning Budde, Davin P. Akko, Herbert E. Ainamani, Eric Murillo-Rodríguez, Roland Weierstall

**Affiliations:** 1 Medical School Hamburg MSH, Department of Psychology, Faculty of Human Sciences, Am Kaiserkai 1, 20457 Hamburg, Germany; 2 Sports Science Department, School of Science and Engineering, Physical Activity, Physical Education, Health and Sport Research Centre (PAPESH), Reykjavik University, Reykjavik, Iceland; 3 Lithuanian Sports University, Kaunas, Lithuania; 4 Department of Psychology and Development Management, Bishop Stuart University, Mbarara, Uganda; 5 Escuela de Medicina, División Ciencias de la Salud, Universidad Anáhuac Mayab Mérida, Mérida, Yucatán Mexico

**Keywords:** Post-traumatic stress disorder (PTSD), Exercise training, Juvenile refugees, Cortisol, Dehydroepiandrosterone (DHEA), Hypothalamic-pituitary-adrenal (HPA) axis

## Abstract

**Background:**

Latest research demonstrates a significant improvement in stress-related symptoms in psychological disorders as a result of exercise training (ET). Controlled clinical trials further validate the significance of ET by demonstrating lower salivary cortisol levels in patients with post-traumatic stress disorder (PTSD) after intervention. A significant change in cortisol and dehydroepiandrosterone (DHEA) levels can already be found after an 8–12-week ET program. The proposed study aims to investigate the impact of an 8-week ET on PTSD symptoms and changes in cortisol levels in a juvenile refugee sample from the Democratic Republic of the Congo (DRC) at an Ugandan refugee settlement. It is the first to implement an ET intervention in a resource-poor, post-conflict setting.

**Methods/design:**

In a randomized controlled trial, 198 adolescent participants aged 13–16 years from the DRC who, suffer from PTSD, will be investigated. The participants are based at the Nakivale refugee settlement, an official refugee camp in Uganda, Africa, which is among the largest in the world. The participants will be randomized into an *Exercise Training (ET) group* with a maximum heart rate (HR_max_) of > 60%, an *Alternative Intervention (AI) group* with low-level exercises, and a *Waiting-list Control (WC) group*. After the 8-week interventional phase, changes in cortisol awakening response (CAR) and DHEA in the ET group that correspond to an improvement in PTSD symptoms are expected that remain at follow-up after 3 months.

**Discussion:**

To date, there is no controlled and reliable longitudinal study examining the effects of an ET program on symptom severity in individuals with PTSD that can be explained with a harmonization of cortisol secretion. The presented study design introduces an intervention that can be implemented with little expenditure. It aims to provide a promising low-threshold and cost-effective treatment approach for the application in resource-poor settings.

**Trial registration:**

German Trials Register, ID: DRKS00014280. Registered prospectively on 15 March 2018.

**Electronic supplementary material:**

The online version of this article (10.1186/s13063-018-2753-x) contains supplementary material, which is available to authorized users.

## Background

With more than 22.5 million refugees worldwide and over 65 million people being forcibly displaced [1], the provision of health services to these populations is among the world’s most significant and challenging endeavors. These numbers are even more alarming when one considers that nine of the top ten hosting countries are not Western countries but mostly low- and middle-income countries [1]. As most of these refugees have had to flee from violent crises and have been exposed to a number of stressful and traumatic experiences, not only in their home countries but also during their flight, millions of them suffer from trauma-related mental health problems, in particular post-traumatic stress disorder (PTSD) [2]. Generally, youth in conflict zones are at risk of developing mental disorders related to their exposure to continuous and traumatic stress, which includes symptoms summarized under the diagnosis of PTSD [3]. Thus, the need for wide-scale, trauma-focused interventions is massively important to restoring refugees’ mental health. The resources that are given, however, are often found to be very low [4] and are particularly scarce for children and adolescents. Therefore, effective steps are required to alleviate the immense human and social costs [5]. Even if psychotherapeutic interventions would be capable of reducing trauma-related suffering [6], most low- and middle-income countries lack a substantial mental health system. Moreover, even if it has been proven that the training of lay counselors and local health workers can be an effective method [7], most professional psychotherapeutic interventions are resource-demanding as they require the application in one-to-one settings [8]. Besides a lack of resources, stigmatization is another big issue for various countries around the world and constitutes another barrier to mental health services, thus contributing to social isolation, distress, and difficulties in the lives of the affected people [9]. The present research initiative, therefore, focuses on Exercise Training (ET) as a promising way to administer cost-effective and low-threshold group-intervention approaches. It will be administered in a refugee settlement in Uganda, a low-income country that is currently the world’s number-eight host country for refugees escaping violent crises in neighboring countries, in particular the Democratic Republic of the Congo (DRC) [1].

### The impact of Exercise Training (ET) on stress-related mental health problems

Exercise Training (ET) has been shown to reduce an individual’s perception of stress and to improve overall mental health [10]. It is thereby important to demarcate exercise from *physical activity*. Both activities can involve large muscle groups that result in substantial increases in heart rate and energy expenditure. The most significant difference between the two is that exercise is planned and structured while physical activity is not [11]. Both can be distinguished as acute and chronic. *Acute exercise* is the physiological response to a single bout of exercise, while *chronic exercise*, which can also be called exercise *training*, refers to the repeated performance of acute exercise [11].

There are several studies describing chronic exercise as a useful intervention for PTSD patients to reduce trauma-related symptoms [12–14]. After implementing an ET program, Fetzner and Asmundson [14] found significantly reduced PTSD symptoms, with 88.9% of the participants reporting a clinically significant improvement. Furthermore, previous research has shown significantly higher reductions in PTSD, depression, anxiety, and stress-symptom severity in PTSD patients who committed to ET, compared to controls [12, 13]. According to a meta-analysis of Rosenbaum et al. [15], who investigated four intervention studies, a higher physical activity was related to less frequent and less-severe PTSD symptoms. Two of these programs used combined aerobic exercises [12, 13], whereas the other two used yoga sessions [16, 17] in their program.

### The glucocorticoid system as a potential target for the beneficial effects of ET

Exercise is a very effective stimulator of the hypothalamic-pituitary-adrenal (HPA) axis [18]. The hypothalamus produces the corticotropin-releasing hormone (CRH) which stimulates the pituitary gland which in turn releases the adrenocorticotropic hormone (ACTH) [19]. ACTH stimulates the adrenal glands to release cortisol and dehydroepiandrosterone (DHEA). DHEA and its sulfate form, dehydroepiandrosterone sulfate (DHEAS), have been found to possess anabolic properties [20], whereas high cortisol has catabolic properties [21]. Furthermore, DHEA/S has neuroprotective and anti-glucocorticoid effects [22]. Under normal conditions DHEA levels are closely correlated with cortisol; however, an imbalance of cortisol/DHEA secretion may occur when an individual experiences chronic stress [23]. Cortisol and DHEA are often addressed as a ratio that represents the balance between anabolic and catabolic hormones [24]. Budde et al. [25] found an increase in cortisol levels due to acute exercise in adolescents. According to Hill et al. [26], the exercise intensity necessary to provoke a significant increase in circulation cortisol is 60% maximum rate of oxygen consumption (VO_2max_), which shows an increase of + 39.9% in circulating cortisol levels. In general, untrained participants had lower VO_2max_ levels, significantly higher responses to cortisol post exercise, and lower hair-cortisol levels compared to participants who exercised regularly [27–29]. In regard to the chronic effects of exercise, it turned out that a higher training volume (measured in kilometers run per week) was associated with an increase in cortisol levels in young healthy men [29]. After ET, a decrease [30] as well as an increase [31] in cortisol levels was found. A distinct rise in cortisol has been observed also immediately after waking, typically peaking 30–45 min after waking [32], and has been appropriately termed the cortisol awakening response (CAR). This response is a neuroendocrine manifestation of the hypothalamic-pituitary-adrenal axis (HPA-axis) has been demonstrated to be sensitive to stressors like exercise. Although normative ranges have been developed for several populations [33], it is still unclear what may constitute a “healthy” cortisol response, as both elevated and depressed responses have been related to dysfunctional psychosocial health status [34]. Compared to adults, fewer data are available on hormone responses to exercise in children and adolescents. Regarding the HPA response to the physical activity status in adolescents, cross-sectional findings revealed no significant changes [35]. However, after presenting an acute exercise-related stressor, young individuals at early stages of puberty showed higher cortisol increases and lower DHEAS-to-cortisol ratios than late pubertal participants [36].

### The significance of glucocorticoids in PTSD

In various studies, alterations in the function of the glucocorticoid system have also been reported for PTSD patients. It is commonly accepted that PTSD patients can have a dysfunction in the HPA-axis [37]. Results on differences in baseline cortisol levels between individuals with PTSD and controls are inconsistent, depending on the research paradigm [38]. Several researchers found reduced basal cortisol levels in PTSD patients compared to healthy controls [39, 40]. In addition, cortisol levels turned out to be a significant predictor for PTSD symptoms 6 weeks and 6 months after the traumatic event [39]. At some time points, even higher cortisol levels were found in participants who had PTSD [41]. Similarly, equivocal results exist surrounding the DHEA response, in particular due to the smaller number of systematic studies in this field. Interestingly, there were negative associations between hair-cortisol levels and the number of different lifetime traumatic events, the frequency of traumatization, and the time interval since traumatization [40]. Regarding the cortisol levels of adolescents and children with PTSD, research reports an increase in cortisol concentration in PTSD victims [42]. Some other studies did not find any significant differences in cortisol levels in this age group [43] or even an attenuation in the CAR [44].

Thus, even if cortisol and DHEA are associated with PTSD and provide promising targets for the detection of changes in HPA functioning, functional consequences related to the development of stress-related psychopathologies will have to be investigated in a subsequent step, constituting a future research avenue. However, the latest studies have demonstrated the usefulness of the present research approach to utilize stress hormones as an objective marker to validate the effectiveness of ET.

Kim and colleagues [45] showed a design that is comparable to the present study by demonstrating significant changes in cortisol levels and also an improvement of PTSD symptom severity after the adult participants completed an 8-week ET program. In their study, “*Mindfulness-based Stretching”* and *“Deep Breath Exercises”* were used as low-intensity chronic exercise interventions. However, the intensity of ET had positive effects on the improvement of PTSD symptoms [12–14].

Even if a few studies suggest ET to be beneficial for children and adolescents with PTSD, no randomized controlled trial (RCT) has been reported yet that directly compares the effects of a cardiovascular condition to a sham condition in a longitudinal design. To our knowledge, there is neither a study investigating the relationship between a chronic exercise intervention and the cortisol response in young PTSD victims, nor has it ever been tested in a resource-poor, post-conflict setting.

### Study aims

The purpose of this paper is to introduce a study protocol for a RCT that aims to systematically investigate the impact of ET on PTSD symptoms and associated cortisol levels in adolescents. It is hypothesized that ET in adolescents not only leads to a sustainable decrease in PTSD symptoms in comparison to two control conditions (H1) but that the changes in PTSD symptoms also correspond to changes in cortisol (H2) and DHEA levels (H3) as markers for beneficial alterations in HPA-axis functioning. Furthermore, we expect differences in PTSD symptom severity and cortisol/DHEA ratios between participants who are highly physically active and those who are less physically active at baseline (t_1_) (H4).

## Methods/design

### Participants

This study investigates juvenile trauma victims from the Democratic Republic of the Congo (DRC), aged 13–16 years, who are based at the Nakivale refugee settlement, an official refugee camp in Uganda, Africa, which is among the largest refugee camps in the world. The government of Uganda and its United Nations High Commissioner for Refugees (UNHCR) partners provide general health services to the refugees in the settlement with few psychotherapeutic interventions. Therefore, Ainamani, Elbert, Olema, and Hecker [46] propose the ample provision of trauma-focused treatment options particularly in these settings in order to support not only individual traumatized refugees but also their families and the whole community. All participants will have a history of traumatic experiences. Participants eligible for inclusion in the intervention only involve individuals with a diagnosis of PTSD according to the *Diagnostic and Statistical Manual of Mental Disorders* (DSM-V). Exclusion criteria cover (1) acute suicidality, acute intoxication, or psychotic symptoms, (2) inability of the parent or legal guardian to provide consent, or (3) child protection issues (e.g., acute maltreatment) that are identified during the initial cross-sectional assessment and are judged by a clinician to make trial inclusion inappropriate. Additionally, the participants may not be part of any other psychological therapy or take any medication with psychoactive drugs until the study is completed. We further control for an unchanged physical activity level during the intervention.

A prior sample size calculation was performed using g*power 3 [47] based on (1) an expected medium effect size in the overall model according to cortisol and PTSD changes reported in previous studies [45], (2) the PTSD symptom severity, the trauma load and the number of potential stressors occurring during the intervention as covariates, and (3) the number of time points. The desired statistical power level was set at 0.95. The sample size calculation was corrected for an estimated dropout rate of 20%, according to other intervention studies that have already been performed in the Nakivale refugee settlement [48]. According to the power analysis, 159 participants are sufficient so that a total sample size of 198 participants is targeted to acknowledge potential dropouts.

### Study design

The present project, which was also registered at www.drks.de before the start of data collection, contains two subsequent steps: (1) a PTSD screening in a large and representative sample of juvenile refugees from the DRC that will serve to identify participants eligible for inclusion in the intervention. A cross-sectional analysis comparing the physical activity levels of the refugees according to their PTSD symptom severity will serve to identify and balance potentially confounding variables. With regard to cross-sectional studies, it is difficult to make causal inferences because it is unknown if the parameter itself (e.g., being cardiovascularly fit) caused the benefit (e.g., reduced symptoms) or if other factors might have accounted for the resultant differences [49]. Therefore, (2) a longitudinal, prospective, and RCT will be implemented covering the ET intervention and two control conditions. *Group* (Exercise Training = ET, Alternative Intervention = AI, and Waiting-list Control = WC) serves as the between-subject factor. Eligible participants will be randomly assigned to one of the three conditions. The participants’ age, their traumatic load indicated by the number of experienced traumatic event types, the baseline PTSD symptom severity, and their physical activity level will serve as matching criteria across groups. The study utilizes a double-blind RCT design.

**Fig. 1:**
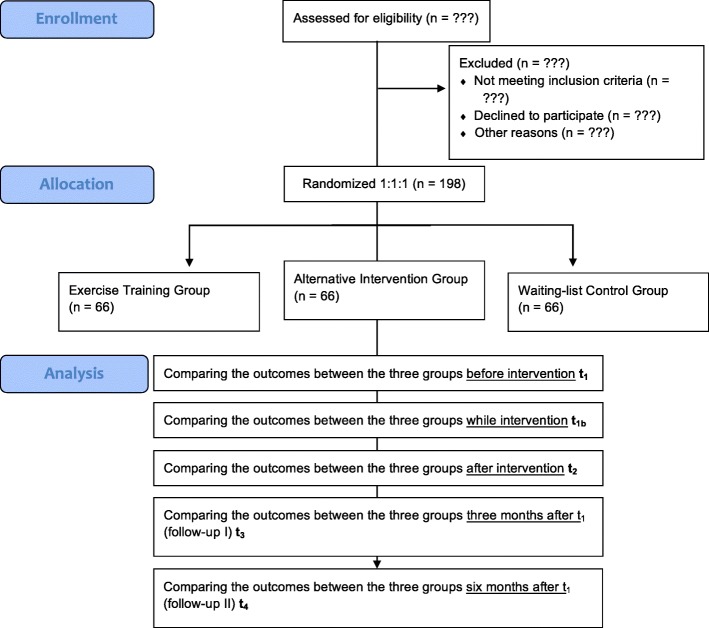
Participant flow diagram

The *ET group* is subjected to an ET of 60–80% of the maximal heart rate (HR_max_). The *AI group* serves as the placebo group and receives fine and gross motor body coordination exercises through playful balance, bilateral coordination, hand-eye coordination, and leg-arm coordination exercises [49]. With such a sham-controlled study, it will be examined whether the expected changes in cortisol levels and symptom severity in PTSD patients are attributed to chronic exercise and no other influencing factors [50]. The *WC group* serves as a no-treatment control and is measured at the same intervals as the treatment group. It will not be implemented until the ET and AI groups have finished their second follow-up (t_4_). Introducing a second control group with the alternative intervention is mandatory to differentiate beneficial effects of miscellaneous physical activity from standardized ET. This is not only important to investigate a potential cross-cultural effectiveness of ET but also serves to justify the specific training requirements of ET and the need for sufficient resources in order to build up sustainable intervention programs. The flow diagram is presented in Fig. 1.

**Fig. 2:**
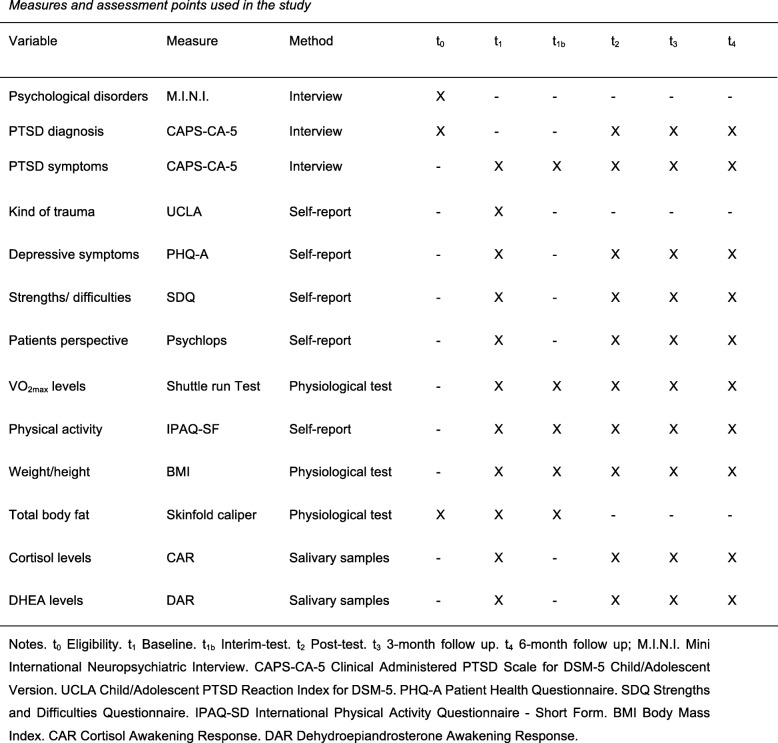
Measures and assessment points used in the study

To assess the impact of chronic exercise on PTSD symptoms and cortisol profiles, repeated measurements with *time* (baseline (t_1_), interim test (t_1b_), post test (t_2_), 3-month follow-up (t_3_), and 6-month follow-up (t_4_)) as the within-subject factor will be conducted on the primary outcome measures *PTSD symptom severity* and awakening *cortisol profiles*. At eligibility (t_0_), the diagnosis will be verified and co-morbid diseases will be identified. In addition, at baseline (t_1_), the PTSD symptom severity, relevant information about the participants’ trauma and flight history, as well as their exposure to early life adversities will be assessed. As physiological measures, cortisol and DHEA levels will be determined. The assignment of participants to one of the three groups will be conducted afterwards. An interim measuring time point (t_1b_) at 4 weeks after the respective intervention will serve to adjust the training to the individual participant’s progress in the two exercise groups in order to assure that the ET stays over 60% HR_max_ after the 8-week interventional phase at the post test (t_2_). The baseline measures will be repeatedly assessed at the post test as well as the two follow-up measuring points (t_3_ and t_4_; see Fig. 2 and Additional file 1).

### Interventions

#### Exercise Training (ET) group

The participants in the ET group will attend three exercise sessions every week for a total number of 8 weeks. That makes this training a chronic exercise intervention [11]. Every unit takes 45 min. Two local and experienced trainers from Uganda instruct identical classes of 25–35 participants. Each session will be performed with high intensity with a HR_max_ of 70–85%. In our preliminary work, significant cortisol and testosterone increases were found by implementing an acute exercise of 70–85% HR_max_, but not with 50–65% HR_max_ [24]. The training program will focus on improvement of cardiovascular fitness through running and running-based games of moderate to vigorous intensity (recorded on three occasions by F1 Polar HR monitors; Polar, Kempele, Finland) [49].

#### Alternative Intervention (AI) group

The aim of this group is to control the placebo effect by implementing a training program in which lower changes in cortisol and DHEA levels, and thereby a lower improvement in PTSD symptoms compared to the ET group, are expected. The participants will attend three 45-min sessions a week for a total number of 8 weeks. The training of the AI group will consist of very little strain, light stretching, and simple exercises that target posture and balance [51] as well as fine and gross motor body coordination through playful balance, bilateral coordination, hand-eye coordination, and leg-arm coordination exercises [49]. These sessions will also be conducted in a group setting by local and experienced trainers.

#### Waiting-list Control (WC) group

No specific intervention will be implemented. However, the ET intervention will be done with this group after the end of the measurement (t_4_).

### Measures

#### Screening

The *UCLA Child/Adolescent PTSD Reaction Index for DSM-5* (UCLA; [52]) is a screening tool recommended by the National Centre for PTSD to collect data about the kind of traumatic happening the participants experienced. It is a 24-item questionnaire assessed to DSM-V criteria for PTSD.

The *Mini International Neuropsychiatric Interview - Kid 7.0.2* (M.I.N.I.; [53]) represents a shortened psychiatric interview on the basis of DSM-V in order to help clinicians diagnose and evaluate the major psychiatric disorders. The test takes about 15–20 min and is performed in form of a standardized interview. The M.I.N.I. will help to identify acute co-morbid diseases relevant for eligibility of the participants.

#### Primary outcome

For the more in-depth baseline PTSD assessment, the *Clinical Administered PTSD Scale for DSM-5 Child/Adolescent Version* (CAPS-CA-5; [54]) will be applied. The CAPS-CA-5 is a 30-item structured interview for the assessment of PTSD based on DSM-5 criteria. The interview will take about 45–60 min and is considered the gold standard for assessing PTSD severity. The CAPS-CA-5 has excellent psychometric properties and has already been successfully administered in other East-African samples [55]. It will serve as the main psychological outcome variable.

#### Secondary outcomes

The Adapted version of the *Patient Health Questionnaire* (PHQ-A; [56]) for the use in adolescent samples will be used as a measure to assess the participants’ depressive symptomatology. It is a brief, nine-item measure that assesses clinically significant symptoms of depressive disorders and episodes in children ages 11–17 years.

The *Strengths and Difficulties Questionnaire* (*SDQ*; [57]) is a brief behavioral screening questionnaire that can serve to identify psychosocial, emotional, and behavioral problems. It is a widely used 25-item measure that has shown its usefulness in various studies and different populations with mental health problems.

The *Psychlops* [58] is a client-generated outcome measure for the assessment of the main psychological problem from the patients’ perspective. It is designed to measure longitudinal changes over the course of an intervention and provides different questions for the baseline, the interim, and the follow-up assessments. In this study, it will also help to capture the most significant mental health problems beyond the diagnosis-specific approach.

#### Hormonal analysis

The determination of the cortisol awakening response (CAR) will take place in following six samples:

1. Sample 1 “awakening” in the morning as soon as the participants wake up
2. Sample 2 “post-awakening I” 30 min after taking the awakening sample
3. Sample 3 “post-awakening II” 45 min after taking the awakening sample.
4. Sample 4 “post-awakening III” 60 min after taking the awakening sample
5. Sample 5 “post-awakening IV” at 9 a.m., collected in the school
6. Sample 6 “post-awakening V” at 11 a.m., collected in the school

Additionally, DHEA levels will be obtained at sample 1, sample 2, and sample 3 only. The samples will be independently collected in salivates by the participants. Saliva will be collected by either passive drooling into the tube or by chewing (for 2 min) the cotton provided with each tube. Participants must wait at least 10 min after smoking, consuming any food or drink, or teeth-brushing before collecting the saliva sample. Previous research has found different stimulation of salivary samples in children with chronic malnutrition [59]. Therefore, our implemented WC group provides the advantage of having a comparable outcome among traumatized children without any treatment condition. In addition, to control the possible influence of malnutrition onto hormonal samples, the Body Mass Index (BMI) and the skinfold thickness will be determined. Tubes will be labeled with patient identifier and returned to the laboratory via collection by the researcher. Upon arrival at the laboratory, tubes with saliva will be centrifuged to remove the upper salivary layer for subsequent measurement of cortisol and DHEA. The DHEA-to-cortisol ratio will be determined by dividing DHEA levels by the respective cortisol levels. The subjects will be informed that the sample has to be collected ± 5 min according to the respective agreed time point. Free cortisol levels in saliva will be measured using a commercially available chemiluminescence assay (IBL, Hamburg, Germany). The evaluation is carried out by C. Kirschbaum in Dresden, Germany.

#### Physical performance measures

In the *Shuttle Run Test* the participants have to run alternately between two lines that are 20 m separated from each other. The running speed will be given by intervals between tone signals. The interval between those signals will be reduced at every level. At the beginning of the test, the running speed is 8 km/h. Every minute it will increase by 0.5 km/h. One minute is roughly equivalent to one level of the shuttle run test. Every time the acoustic hint sounds, the participants have to reach the line. When a subject does not reach the line twice in a row, the test will be over for them. The graduated mileage will be noted and the appropriate VO_2max_ level can be read off [25].

The *International Physical Activity Questionnaire - Short Form* (IPAQ-SF; [60]) captures the general physical activity level of individuals by calculating the time of moderate and vigorous physical activity as well as the time that the participants spend walking and sitting. The questionnaire had also been used in an African context [61] and refers to the last 7 days.

The BMI will also be calculated by taking the body weight in kilograms and dividing it by the height [62].

The total body fat will be determined by using a skinfold caliper.

A detailed list of all measuring instruments and the times points at which they will be assessed is given in Fig. 2.

#### Assessment

Due to the expected low experience with mental health-related questions in the target populations, all psychological instruments will be administered as semi-structured clinical interviews held in Kiswahili. The study and clinical interviews will be conducted by a team of experienced master of science students (psychology) from both Mbarara University and Bishop Stuart University in southwestern Uganda. The research team will be extensively trained in the concepts of mental disorders, data collection, and interviewing techniques for a period of 3 weeks, both through in-person training and regular Skype meetings. Additionally, trained, bilingual, English-speaking local interpreters will be trained alongside experienced clinical and counseling psychology students in clinical interviewer skills to support the assessments. This procedure has already been successfully used in other projects of HA and RW and has proven its validity in various mental health studies in East African samples. The team of investigators will also receive constant supervision by HA and RW, both clinical psychologists/psychotherapists with extensive experience in the conduction of studies in sub-Saharan post-conflict settings.

The psychological measures have already been successfully applied in other refugee samples in resource-poor settings by HA and RW. The translation process for those measures that have not yet been translated into Kiswahili involves forward translations by two bilingual clinical psychologists, synthesis, back translation by two different experts, comparison and agreement on translation and review, and modification by a team of local experts, local assistants from the refugee community, and the principal investigators (PIs). The procedure also involves a piloting program involving juvenile DRC refugees to identify problematic phrases in the informed consent form, the instructions, and the items. This translation procedure is commonly used in studies focusing on mental health issues in similar samples and has proven its validity.

#### Initial procedure

This study will purposively select 198 refugee adolescents using the quota sampling procedure that considers the proportions of different sexes. Hence, the sample will constitute 99 female and 99 male adolescent refugees each. Every third household in each selected zone will be selected as the target household until the desired number of participants is reached. Before the interview, content, procedure, risks, the right to withdraw, and confidentiality will be explained to each participant and written informed consent (signature or fingerprints in case of illiteracy) will be obtained. In addition to their parents, children, and adolescents will also be asked to give their informed consent. Potential participants as well as their legal authorized representatives will be fully informed about the study aims, the possible inconveniences of the clinical interview procedures, the compensation for participation, and the eligibility conditions of the treatment trial. After obtaining informed consent, participants will be screened regarding (1) their PTSD symptom severity, (2) potential exclusion criteria, in particular suicidality and psychotic symptoms according to the M.I.N.I., and (3) child protection issues according to their reports in the trauma event list and their clinical appearance during their contact with the care-takers and the interviewers. Participants with noticeable problems will receive a second check up by one of the PIs and will receive referrals if action is required. The screening will take about 60 min. Participants who are eligible for inclusion in the treatment trial will attend a more in-depth assessment that will take about 80 min. Afterwards, they will be randomly assigned to one of the three interventions.

#### Statistical analysis

Over the course of the intervention, we will utilize a repeated-measure, mixed-model approach in order to identify significant changes in the main outcome variables between the three groups. Differences in baseline PTSD symptom severity will be considered to acknowledge nonlinear treatment effects between participants with a different burden at t_1_. Potential confounding variables, such as the exposure to potentially traumatic events during the course of the intervention, will be used as covariates. All analyses will be performed using R statistics and MPlus.

#### Methods to protect against sources of bias

All participants who were not excluded through allocation will be randomized by a computer-generated program into the ET, AI, and WC groups. Furthermore, the interviewers do not know to which group the participants belong. All participants are asked to not talk about their intervention at any time point. The trainers are also blinded regarding the hypotheses of the researchers and the intervention under investigation. To acknowledge potential effects of allegiance, trainers are not informed about expected differences in the effectiveness of the interventions. Data analysts are blinded to group allocation. Blindness will be set aside when a participant is (1) suicidal, (2) acutely in danger, (3) overwhelmed by the intervention, (4) develops an additional psychological disorder, (5) loses a massive amount of weight, or (6) suffers under malnutrition. All names will be pseudonymized and anonymized and can only be undone by HB. Furthermore, 15% of the CAPS-CA-5 interviews are reassessed by a second interviewer to establish inter-rater reliability of the PTSD diagnosis and symptom severity.

#### Ethics approval and consent to participate

The Ethical Review Boards of the Medical School Hamburg approved the study. Additionally, further permission will be sought from the Uganda National Council for Science and Technology. Informed consent will be sought from each prospective participant and the participant’s legally authorized representative. If any participant is likely to be susceptible to undue influence or coercion, the investigators will enforce additional safeguards in the study to protect such participants. Additional referrals will be offered to patients suffering from acute suicidality or other issues that require acute treatment. As the provision of mental health services in the refugee settlement is sparse we shall refer patients with acute mental health problems to Mbarara Hospital Referral Department of Psychiatry for further treatment. Each participant of the ET, AI, and WC groups will receive 20,000 Uganda shillings, equivalent to US$7, for compensation of their time and participation. In case of a significantly positive treatment effect, additional ET will be offered to participants from the AI and WC groups after completion of the study to ensure access to a beneficial intervention. Potential study participants and their parents must provide written informed consent before they can be included in the study. Personal information about potential and enrolled participants will be collected and stored separately from other study data and will be only accessible for the assessors who contact the participants for data assessment. The study dataset does not include personal information and will be analyzed by the coordinating study site. In case of an unexpected serious adverse event (e.g., life-threatening event, permanent damage, or death) over the course of the study, the coordinating site will alert the PI who will report the serious adverse event to the local Ethics Committee. The Ethics Committee and the study team will then decide in accordance with the best interest of the patient if the study procedures are continued or terminated.

## Discussion

This study is designed to investigate the effects of an ET program on the cortisol levels and symptoms of young individuals with PTSD. There have been previous trials examining the relationship between exercise and PTSD, that have found significant improvements in symptom severity through ET [12–14]. In regard to the relationship between cortisol and PTSD, most studies confirmed lower cortisol levels in PTSD patients [39, 40, 63], even though in some cases higher cortisol levels were found [38]. The relationship between cortisol and exercise in healthy subjects, confirms changes through an implementation of an ET, but the response of cortisol varied in those studies and depended on age [18, 30, 31, 35], showing no different secretion due to the physical activity status for adolescent students [35]. The aim of the described study is a combination of three factors: ET, PTSD symptom severity, and cortisol/DHEA. There is only one related study investigating this subject, using a *Mindfulness-based Stretching* intervention in an adult sample. Those researchers found significant changes in cortisol levels and also an improvement in PTSD symptom severity after the participants completed the 8-week ET program. They confirmed that improved PTSD scores were associated with a normalization of cortisol levels [45]. It is unclear whether this intervention will be intense enough for inducing cortisol changes in the present younger population [25]. since a higher exercise intensity is needed to induce changes in cortisol levels after an ET program [26]. Taken together, PTSD patients have a dysfunction in the HPA-axis [37] and we expect the cortisol levels of PTSD patients to change by implementing an ET program. Furthermore, we assume that the change in cortisol levels will be associated with an improvement of the PTSD symptoms.

The present study introduces an interventional program that is easy to implement and, compared to most psychological interventions, has the advantage of being administered in groups. It is generally accepted that group interventions that can be administered by laymen are more cost-effective than therapeutic interventions in a one-to-one setting that require extensive psychotherapeutic expertise. However, besides face validity, the cost-effectiveness of an ET intervention as proposed in the present publication has not yet been evaluated in terms of direct and indirect costs. Thus, the present study provides further promising targets for interdisciplinary research on economic aspects of different potential health services in refugee settings.

Another advantage is that ET is not difficult to introduce and no additional costly materials are needed. This is in particular important for an ample provision of interventions in resource-poor settings. Another benefit of the present work lies in the recruitment of the participants in the refugee settlement in Uganda, Africa, who are highly affected by PTSD [64]. Previous research that strictly used cross-sectional study designs offers a limited explanation for the aim of the present work. Therefore, in addition to a cross-sectional program, a longitudinal study design was planned. This might provide the research field with more meaningful and confirmatory results. In sum, there is no study regarding the effect of an ET program on cortisol levels and symptom severity in adolescent individuals with PTSD. Therefore, the presented longitudinal RCT will close this gap and will allow for a new promising approach to overcome trauma-related suffering in PTSD-affected refugee populations.

### Trial status

TRLS-D-18-00245R, 18 June 2018, no recruitment yet.

## Additional file

Additional file 1: Standard Protocol Items: Recommendations for Intervention Trials (SPIRIT) 2013 Checklist: recommended items to address in a clinical trial protocol and related documents. (PDF 71 kb)

## Abbreviations

ACTH: Adrenocorticotropic hormone
AI: Alternative Intervention
BMI: Body Mass Index
CAPS-CA-5: Clinical Administered PTSD Scale for DSM-5 Child/Adolescent Version
CAR: Cortisol awakening response
CRH: Corticotropin-releasing hormone
DHEA: Dehydroepiandrosterone
DHEAS: Dehydroepiandrosterone sulfate
DSM: Diagnostic and Statistical Manual of Mental Disorders
ET: Exercise training
HPA-axis: Hypothalamic-pituitary-adrenal axis
HR_max_: Maximal heart rate
IPAQ-SF: International Physical Activity Questionnaire - Short Form
M.I.N.I: Mini International Neuropsychiatric Interview
PHQ-A: Patient Health Questionnaire
PTSD: Post-traumatic stress disorder
RCT: Randomized controlled trial
SDQ: Strengths and Difficulties Questionnaire
UCLA: Child/Adolescent PTSD Reaction Index for DSM-5
UNHCR: United Nations High Commissioner for Refugees
VO_2_: Volume of oxygen
VO_2max_: Maximum rate of oxygen consumption
WC: Waiting-list control
